# Oregano essential oil modulates colonic homeostasis and intestinal barrier function in fattening bulls

**DOI:** 10.3389/fmicb.2023.1293160

**Published:** 2023-12-05

**Authors:** Yue Ma, Jinping Shi, Li Jia, Pengjia He, Ying Wang, Xiao Zhang, Yongliang Huang, Qiang Cheng, Zhao Zhang, Youchao Dai, Meiling Xu, Zhaomin Lei

**Affiliations:** ^1^College of Animal Science and Technology, Gansu Agricultural University, Lanzhou, China; ^2^Gansu Xu Kang Food Co., Ltd., Pingliang, China; ^3^Gansu Huarui Agriculture Co., Ltd., Zhangye, China

**Keywords:** oregano essential oil, colonic microbiota, short-chain fatty acids, microbial metabolites, intestinal barrier function, fattening bulls

## Abstract

Oregano essential oil (OEO) primarily contains phenolic compounds and can serve as a dietary supplement for fattening bulls. However, the precise molecular mechanism underlying this phenomenon remains largely elusive. Therefore, this study investigated the impact of adding OEO to diet on the integrity of the intestinal barrier, composition of the colonic microbiome, and production of microbial metabolites in fattening bulls. Our goal was to provide insights into the utilization of plant essential oil products in promoting gastrointestinal health and welfare in animals. We employed amplicon sequencing and metabolome sequencing techniques to investigate how dietary supplementation with OEO impacted the intestinal barrier function in bulls. The inclusion of OEO in the diet resulted in several notable effects on the colon of fattening bulls. These effects included an increase in the muscle thickness of the colon, goblet cell number, short-chain fatty acid concentrations, digestive enzyme activity, relative mRNA expression of intestinal barrier-related genes, and relative expression of the anti-inflammatory factor IL-10. Additionally, α-amylase activity and the relative mRNA expression of proinflammatory cytokines decreased. Moreover, dietary OEO supplementation increased the abundance of intestinal *Bacteroides*, *Coprobacillus*, *Lachnospiraceae_UCG_001*, and *Faecalitalea*. Metabolomic analysis indicated that OEO primarily increased the levels of 5-aminovaleric acid, 3-methoxysalicylic acid, and creatinine. In contrast, the levels of maltose, lactulose, lactose, and D-trehalose decreased. Correlation analysis showed that altered colonic microbes and metabolites affected intestinal barrier function. Taken together, these results demonstrate that OEO facilitates internal intestinal environmental homeostasis by promoting the growth of beneficial bacteria while inhibiting harmful ones.

## 1 Introduction

The colon serves as a crucial organ for fermentation and absorption in the hindgut of ruminants. Within the intestinal lumen, a complex symbiotic microbiota plays a vital role in the host’s health through complex biological functions and metabolic processes (Lin et al., 2021). Although the hindgut plays a smaller role in digestion and metabolism than the rumen in ruminants, hindgut microbes can still utilize more than 10% of the dietary carbohydrates. Moreover, the hindgut adaptively responds to increased amounts of organic material and promotes fermentation (Gressley et al., 2011). In the hindgut of ruminants, microbes digest up to 15–30% of hemicellulose (Dixon and Nolan, 2005), and 8–17% of total short-chain fatty acids are absorbed (Hoover, 1978). This contributes to the metabolic energy needs of cattle (Váradyová et al., 2000) and sheep (Faichney, 1968) by 5–12 and 10%, respectively.

The intestinal epithelium acts as a functional mediator between symbiotic microbes and host animals, regulating intestinal defense, metabolism, and immunity through a series of cellular molecules. The intestinal barrier includes various symbiotic bacteria, cells, and soluble substances (Pan et al., 2019) and can be classified as a physical, chemical, immune, or microbial barrier. The intestinal barrier is an important pathway through which the intestinal epithelium effectively prevents the invasion of toxic and harmful materials (Suzuki, 2020). In contemporary livestock production, there have been significant advancements in bull-fattening techniques. However, high-grain diets, commonly used for prolonged periods to fatten animals to achieve better performance (Gao and Oba, 2014), can cause significant damage to the intestinal barrier. The intestinal microbiota contributes to the digestion of food, metabolizes indigestible components, and produces metabolites that regulate host health and immune defense (Maurice et al., 2013). Among ruminants, the colon in the hindgut is more susceptible to dietary changes than the rumen (Lin et al., 2021). Consequently, modulating the hindgut microbiota and its metabolites through dietary modulation can reduce inflammation, stabilize the microbiota, and improve intestinal barrier function (Lin and Zhang, 2017), which may be an effective approach to improving host health.

Plant secondary metabolites, specifically essential oils, are recognized as promising alternatives to many antibiotic candidates (Nalle and Turner, 2015) and are widely used in livestock production (Zhang et al., 2021). Oregano essential oil (OEO) is a plant-derived feed additive whose primary active ingredients include phenolic substances, such as thymol (2-isopropyl-5-methylphenol) and carvacrol (5-isopropyl-2-methylphenol), which have broad-spectrum antibacterial and antioxidant properties (Froehlich et al., 2017). The hydroxyl group of phenols regulates apoptosis and exerts antimicrobial activity by damaging bacterial cell membranes, causing the leakage of intramembrane substances (Lv et al., 2011). An *in vitro* study found that OEO not only increased the permeability of *Staphylococcus aureus* and *Pseudomonas aeruginosa* cell membranes (Lambert et al., 2001) but also effectively controlled the levels of *Alicyclobacillus* (Dutra et al., 2019). OEO is frequently used in rumen production, and its use as a food supplement has been shown to improve cattle rumen digestion and growth performance (Zhang et al., 2021). This results in enhanced weight gain in cattle, translating into greater earnings and more economic benefits. Moreover, OEO can modulate sheep’s small intestine microbiota to promote growth (Jia et al., 2022). Furthermore, OEO can replace antibiotics in lamb diets, positively impacting meat quality (Garcia-Galicia et al., 2020). These studies suggest that OEO can modulate the composition of the gastrointestinal microbiota, benefiting the product. However, the precise molecular mechanisms for effectively regulating product quality through changes in ruminant gastrointestinal microbes remain poorly understood.

Previous studies have primarily focused on the influence of OEO on livestock carcasses, meat quality, and rumen digestibility. However, the impact of OEO on livestock host physiology, intestinal homeostasis, and barrier function remains poorly understood. Our study addressed this gap. We postulated that including OEO in the diet would alter the colonic microbiota of Holstein fattening bulls. We further hypothesized that these alterations would subsequently impact microbial metabolite production in the colon and influence the integrity of the intestinal barrier, improving the host’s overall health. We used amplicon sequencing and metabolomics to determine how OEO affects the colonic microbiota of Holstein fattening bulls, providing a theoretical and practical foundation for using OEO as a plant-derived feed additive to maintain intestinal health.

## 2 Materials and methods

### 2.1 Animals and experimental design

A total of 18 10-month-old Holstein bulls with similar initial average body weights (345.19 ± 3.89 kg) were selected for the experiment. They were randomly divided into two groups of nine animals each. All bulls were housed individually. The control group (CON) was fed a basal diet, whereas the OEO group was fed a basal diet supplemented with 20 g/(d·head) of OEO (Ralco Inc., Marshall, MN, USA). The fattening trial spanned 300 days, which consisted of a 30-day acclimation period, followed by 270 experimental days. The diet, primarily corn silage and grain mixtures, was adjusted every 30 days (Supplementary Table S1) to meet or exceed beef cattle nutrient requirements as specified by the National Research Council (NRC 2016).

### 2.2 Sample collection

At the end of the fattening experiment, power analysis based on the final body weights of fattening bulls (CON = 682.68 ± 35.64. OLE = 762.63 ± 59.39, α = 0.05) resulted in an actual power (1-β) of 0.72 when the sample size was six (*n* = 6) in each group. A total of six bulls were randomly selected from each group for killing. The colonic contents were collected from the mid-colon and divided into two sections. One section, containing approximately 12 mL, was snap-frozen in liquid nitrogen and stored at −80°C for later analysis using metabolomic and 16S rRNA gene sequencing techniques. Approximately 15 mL of colonic contents from the other section was stored at −20°C for subsequent measurement of colonic digestive enzyme activity (Jia et al., 2022). Colonic tissues from the central parts were split into two parts. For intestine histomorphological investigation, one part was fixed in 4% paraformaldehyde. The second part was frozen in liquid nitrogen for mRNA expression analysis.

### 2.3 Colonic histomorphology and goblet cell numbers

The morphological structure of the colon was observed under a light microscope (LEICA-DM400; Leica, Germany) after hematoxylin and eosin staining. Photographs were taken using a digital pathology system (DX1; 3DHISTECH, Budapest, Hungary). Ten microscopic fields were randomly selected per sample to measure colonic muscle thickness using Pannoramic Viewer software, version 1.15.3 (3DHISTECH). Goblet cells were visualized using the periodic acid-Schiff staining procedure (Yamabayashi and Tsukahara, 1987) and counted in 10 fields per bull. In summary, one technical replicate of each biological replicate was used to observe the contents under 10 fields of view.

### 2.4 pH, fermentation parameter, and digestive enzyme activity measurement

The pH of the colonic contents was measured immediately using an Ark Technology PHS-10 portable acidity meter (Chengdu, China) by mixing 1 mL of phosphoric acid (0.5% v/v) solution with 20 mg of colonic contents. Colon content samples were uniformly ground. After pretreatment, SCFAs contents were detected by MetWare^1^ based on the Agilent 7890B-7000D GC–MS/MS platform. The homogenate mixture and colonic contents were sonicated, and the resulting 10% homogenization buffer’s supernatant was collected. The enzyme activities in the samples were determined using standard kits according to the manufacturer’s instructions (Nanjing Jiancheng Bioengineering Institute, Nanjing, China). Enzyme activities (β-glucosidase, cellulase, α-amylase, lipase, and xylanase) were determined using colorimetric assays (Zhang et al., 2021). Pectinase levels were determined using a micromethod.

### 2.5 Quantitative real-time PCR analysis

Total RNA was isolated from each sample using TRIzol reagent (Invitrogen, USA). RNA concentration was detected using a NanoDrop2000 (Thermo Fisher Scientific, USA). The PrimeScript 1st Stand cDNA Synthesis Kit (Takara Biomedical Technology Co. Ltd., Beijing, China) was used to reverse transcribe RNA into cDNA. The reaction system comprised 20 μL. The reaction procedures were as follows: 95°C for 5 min; 40 cycles of 95°C for 15 s, 60°C for 30 s. *GADPH* was used as an endogenous control, and the relative expression of genes was calculated using the 2^−ΔΔCt^ method (Livak and Schmittgen, 2001). The primer sequences are listed in Table 1.

**Table 1:** Primer information.

Target gene^1^	Primer sequence (5′-3′)^2^	Product size (bp)	Accession number
*GAPDH*	F: GCACAGTCAAGGCAGAGAAC R: CATACTCAGCACCAGCATCAC	110	ENSBTAG00000014731
*Occludin*	F: CTCGCTGCCATTACCTGAA R: CGCTGTCTCCTTGGTCATC	77	ENSBTAG00000011135
*Claudin-1*	F: GCATCCTGCTGGGACTAAT R: TACACTTCATGCCAACGGT	53	ENSBTAT00000017476
*Claudin-4*	F: TATGGATGAACTGCGTGGTG R: GCCAGGATGATACAGATGACG	123	ENSBTAG00000026278
*ZO-1*	F: ATGTTTATCGTCGCATCGT R: CGTTCCACCTCTTTATGGTT	198	ENSBTAG00000015398
*MUC-1*	F: CTGCTGTTCCCAGTGCTTAC R: GGGCTGCTTTGTGTAGTGG	103	ENSBTAG00000017104
*MUC-2*	F: TACCGAAGCAGATGGAGATG R: GCAGAGGAGTGTTGGGAAA	103	XM_024987595.1
*TNF-α*	F: TCTTCTGCCTGCTGCACTT R: GGGCTACCGGCTTGTTACT	131	NM_173966.3
*TLR-4*	F: AGATTTGTCCTTGAACCCTTT R: TAAACCAGCCAGACCTTGA	135	ENSBTAG00000006240
*IL-1β*	F: GTGCAAACTCCAGGACAGA R: ACACCACTTCTCGGTTCATT	103	ENSBTAG00000001321
*IL-6*	F: TGACTTCTGCTTTCCCTACC R: CCTTGCTGCTTTCACACTC	137	ENSBTAG00000014921
*IL-10*	F: CGTGACCTCCATCCACTCT R: GGCAGGGAGCAGTCATTTA	187	ENSBTAG00000006685

^1^GAPDH, glyceraldehyde-3-phosphate dehydrogenase; ZO-1, zonula occluden-l; MUC-1, mucoprotein-l; MUC-2, mucoprotein-2; TNF-α, tumor necrosis factor-α; TLR-4, toll-like receptor 4; IL-1β, interleukin-1β; IL-6, interleukin-6; IL-10, interleukin-10. ^2^F, forward primer; R, reverse primer.

### 2.6 ZO-1 and MUC-2 measurement using immunohistochemistry

Immunohistochemistry was performed using specific rabbit and human monoclonal antibodies to analyze the distribution of colonic ZO-1 and MUC-2 expression in bulls. Anti-ZO-1 (bs-1329R; Bioss, China) and anti-MUC-2 antibodies (Clone ABT198; Immunoway, USA) were used for immunohistochemistry assays.

### 2.7 16S rRNA gene sequencing of the colonic microbiota

Microbial DNA was isolated from 12 colonic samples using a HiPure Soil DNA Kit (Magen, Guangzhou, China) according to the manufacturer’s instructions. Primers 341F (5′-CCTACGGGNGGCWGCAG-3′) and 806R (5′-GGACTACHVGGGTATCTAAT-3′) were amplified using the V3–V4 region of bacterial 16S rDNA (Guo et al., 2017). The 16S rDNA target region of the ribosomal RNA gene was amplified using PCR.

Amplicons were extracted from 2% agarose gels, purified using an AxyPrep DNA Gel Extraction Kit (Axygen Biosciences, Union City, CA, USA) according to the manufacturer’s instructions, and quantified using the ABI StepOnePlus Real-Time PCR System (Life Technologies, Foster City, CA, USA). The raw reads were deposited in the NCBI Sequence Read Archive database (Accession Number: PRJNA995187). FASTP (version 0.18.0) filtered raw readings for clean reads (Chen et al., 2018). FLASH (version 1.2.11) integrated paired-end clean readings as raw tags with a 10 bp overlap and 2% mismatch error rate (Magoč and Salzberg, 2011). The noisy raw tag sequences were filtered under particular circumstances to produce clean tags (Bokulich et al., 2013). The UPARSE (version 9.2.64) pipeline grouped clean tags into operational taxonomic units with ≥97% similarity. All chimeric tags were removed using the UCHIME algorithm (Edgar et al., 2011), and effective tags were obtained for further analysis.

### 2.8 Metabolite profiles of colonic contents

The sequencing technique was the same as our previous study (Jia et al., 2022). Sample extracts were analyzed using an LC-ESI-MS/MS system (UPLC, ExionLC AD^2^; MS, QTRAP® System). LIT and triple quadrupole (QqQ) scans were acquired on a triple quadrupole-linear ion trap mass spectrometer (QTRAP) QTRAP® LC–MS/MS System and controlled by Analyst 1.6.3 software (Sciex). Instrument tuning and mass calibration were performed using 10 and 100 mol/L polypropylene glycol solutions in the QqQ and LIT modes, respectively. A specific set of MRM transitions was monitored for each period according to the metabolites eluted within this period.

### 2.9 Data statistics and analysis

Histology, fermentation parameters, digestive enzyme data, and relative gene expression data were analyzed using independent sample *t*-tests in SPSS (version 27.0). Data are presented as the mean ± SEM. GraphPad Prism (version 9.2) was used to generate the statistical maps.

For microbial community profiling, alpha diversity analysis, including Chao1 and Simpson indices, was performed based on the Wilcoxon rank-sum test. Principal coordinate analysis based on binary-hamming distances was performed to analyze the similarities or differences in the compositions of the bacterial communities. Linear discriminant analysis effect size (LEfSe) analysis was performed using LEfSe software, which outputs linear discriminant analysis (LDA) histograms (LDA score > 3.0) and cladograms. Functional contributions of the intestinal microbiota were assessed using the PICRUSt2 tool.

Metabolome statistics were analyzed using MultiQuant software. Orthogonal projections to latent structure-discriminant analysis (OPLS-DA) was used to determine metabolic differences between the two groups. Differential metabolite screening was performed for variable important in projection (VIP) ≥ 1.0 and fold change (FC) ≥ 1.5 or ≤ 0.67. The Kyoto Encyclopedia of Genes and Genomes (KEGG) database was used to annotate the results for enrichment and classification of pathways. Spearman’s correlation coefficient was analyzed using SPSS version 27.0, and a heatmap of Spearman’s correlation analysis, two-way orthogonal partial least squares (O2PLS) analysis, and a network heatmap were generated using OmicShare Tools.

## 3 Results

### 3.1 Colonic histomorphology and goblet cell numbers

**Figure 1:**
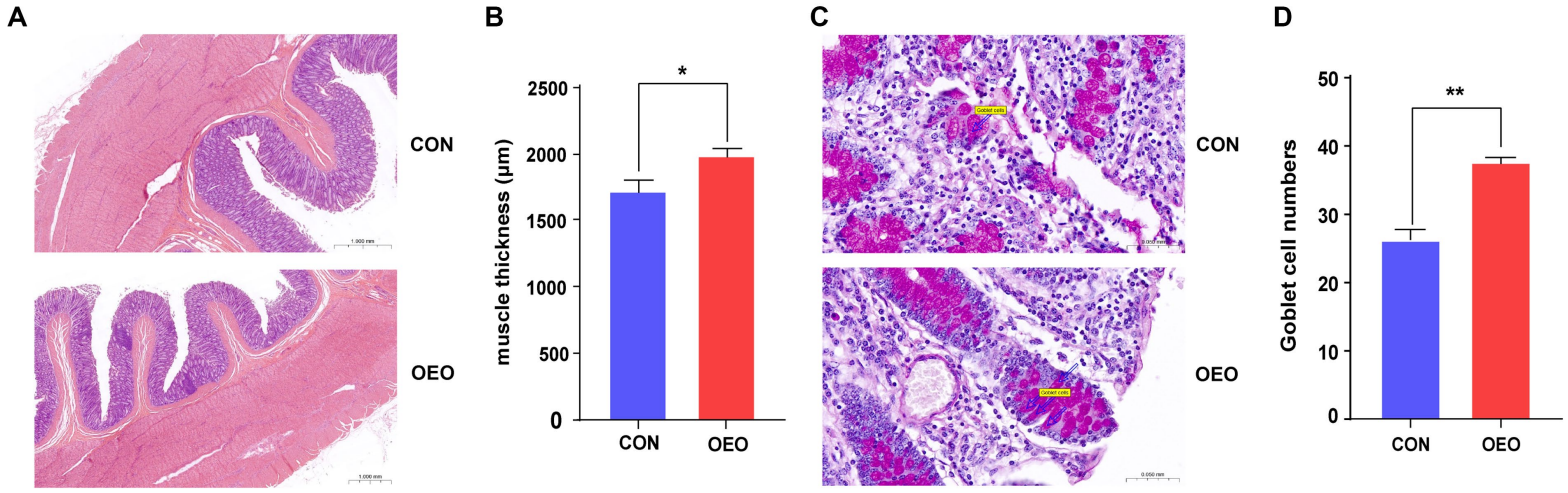
Effects of OEO on the colonic histomorphology and goblet cell numbers. **(A)** Intestinal morphology of colon in bulls. **(B)** Muscle thickness of the colon. **(C)** Distribution of goblet cells in the colonic epithelium. The markers in the picture represent goblet cells. **(D)** Colonic goblet cell numbers. *n* = 6 samples/group. Significance is reported as *p < 0.05, **p < 0.01.

Dietary supplementation with OEO reduced the space between colonic epithelial cells and resulted in a tendency for repair and thickening (Figure 1A). Colon thickness in the OEO group was significantly greater than that in the CON group (*p* < 0.05) (Figure 1B). The inclusion of OEO in the diet significantly increased the number of goblet cells in the colonic epithelium compared to the CON group (*p* < 0.01) (Figures 1C,D). These results suggest that OEO supplementation improves colonic morphology.

### 3.2 Colonic fermentation parameters and digestive enzyme activities

The pH of the colonic contents was not significantly different between the two groups. The concentrations of propionic and butyric acids were significantly higher in the OEO group than in the CON group (*p* < 0.05); however, the difference in acetic acid concentration was not significant (*p* > 0.05). Moreover, the α-amylase activity was significantly lower in the OEO group than in the CON group (*p* < 0.01). However, the β-glucosidase, lipase, xylanase, cellulase, and pectinase activity rates were significantly higher in the OEO group than in the CON group (*p* < 0.01) (Table 2). Overall, these results suggest that OEO supplementation is beneficial for colonic fermentation and digestibility.

**Table 2:** Effects of OEO on the pH, fermentation parameters, and digestive enzyme activities in the colonic contents.

Items	Groups		SEM	*p*-value
	CON	OEO		
pH	7.05	6.88	0.13	0.217
Acetic acid (mg/g)	2.21	2.89	0.30	0.051
Propionic acid (mg/g)	0.57	0.74	0.06	0.023
Butyric acid (mg/g)	0.24	0.35	0.04	0.037
α-amylase (U/g·protein)	25.95	11.55	4.18	0.006
β-glucosidase (U/g·protein)	80.99	148.90	8.99	0.000
Lipase (U/g·protein)	26.06	45.13	1.44	0.000
Xylanase (U/g·protein)	79.71	161.10	6.21	0.000
Cellulase (U/g·protein)	16.56	36.16	2.34	0.000
Pectinase (U/g·protein)	37.55	67.05	3.47	0.000

### 3.3 Relative mRNA expression of intestinal barrier-related genes

**Figure 2:**
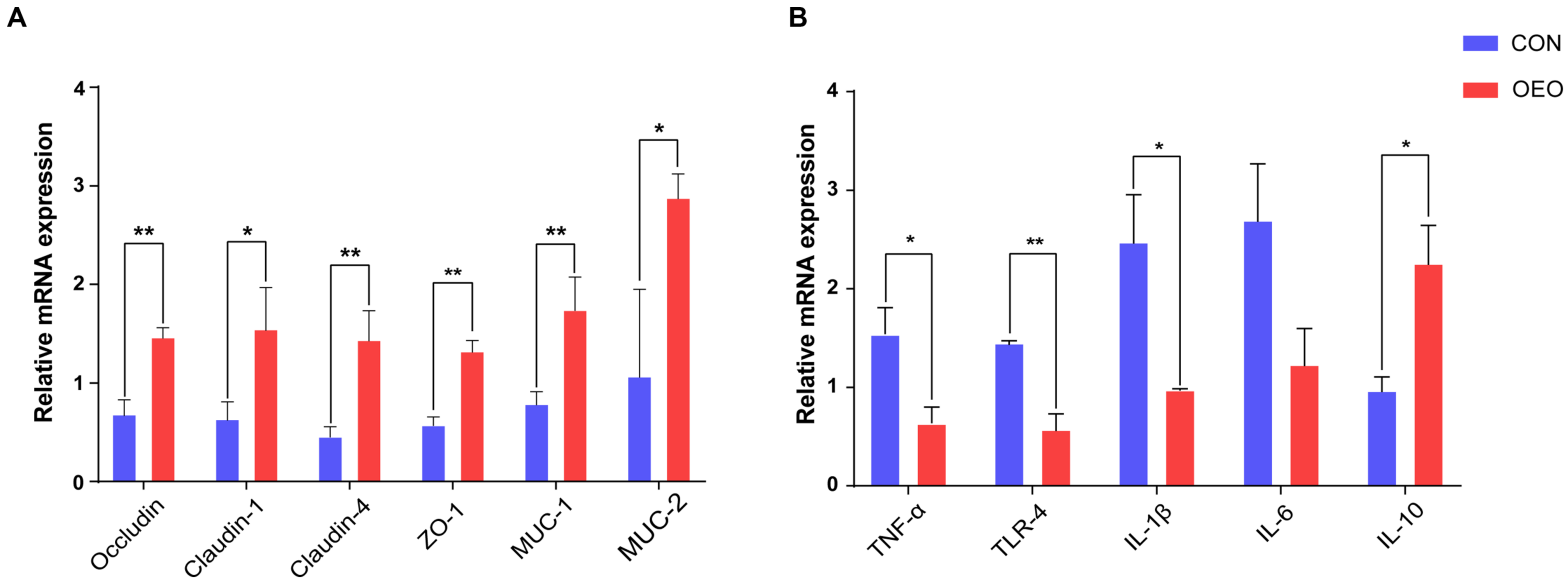
Effects of OEO on the relative mRNA expression of intestinal barrier-related genes. **(A)** Relative mRNA expression of genes related to tight junction proteins and mucin proteins. **(B)** Relative mRNA expression of genes related to cytokines. *p < 0.05, **p < 0.01.

To assess the influence of OEO on the intestinal barrier, the expression of tight junction proteins, mucins, and inflammatory cytokines in the colonic epithelium was measured using quantitative real-time PCR. The mRNA expression of *Claudin-1*, *MUC-2* (*p* < 0.05), *Occludin*, *Claudin-4*, *ZO-1*, and *MUC-1* (*p* < 0.01) significantly increased (Figure 2A), while that of proinflammatory cytokines *TLR-4* (*p* < 0.01), *TNF-α*, and *IL-1β* (*p* < 0.05) significantly decreased (Figure 2B) in the OEO group compared with that in the CON group. Moreover, the mRNA expression of the inflammatory cytokine *IL-10* significantly increased (*p* < 0.05). These results suggest that OEO supplementation increases the expression of intestinal tight junction proteins and mucin-related genes in the colonic epithelium and reduces colonic inflammation, thereby enhancing the colonic barrier function of fattening bulls.

### 3.4 Positive expression location of ZO-1 and MUC-2 proteins in colonic epithelium

**Figure 3:**
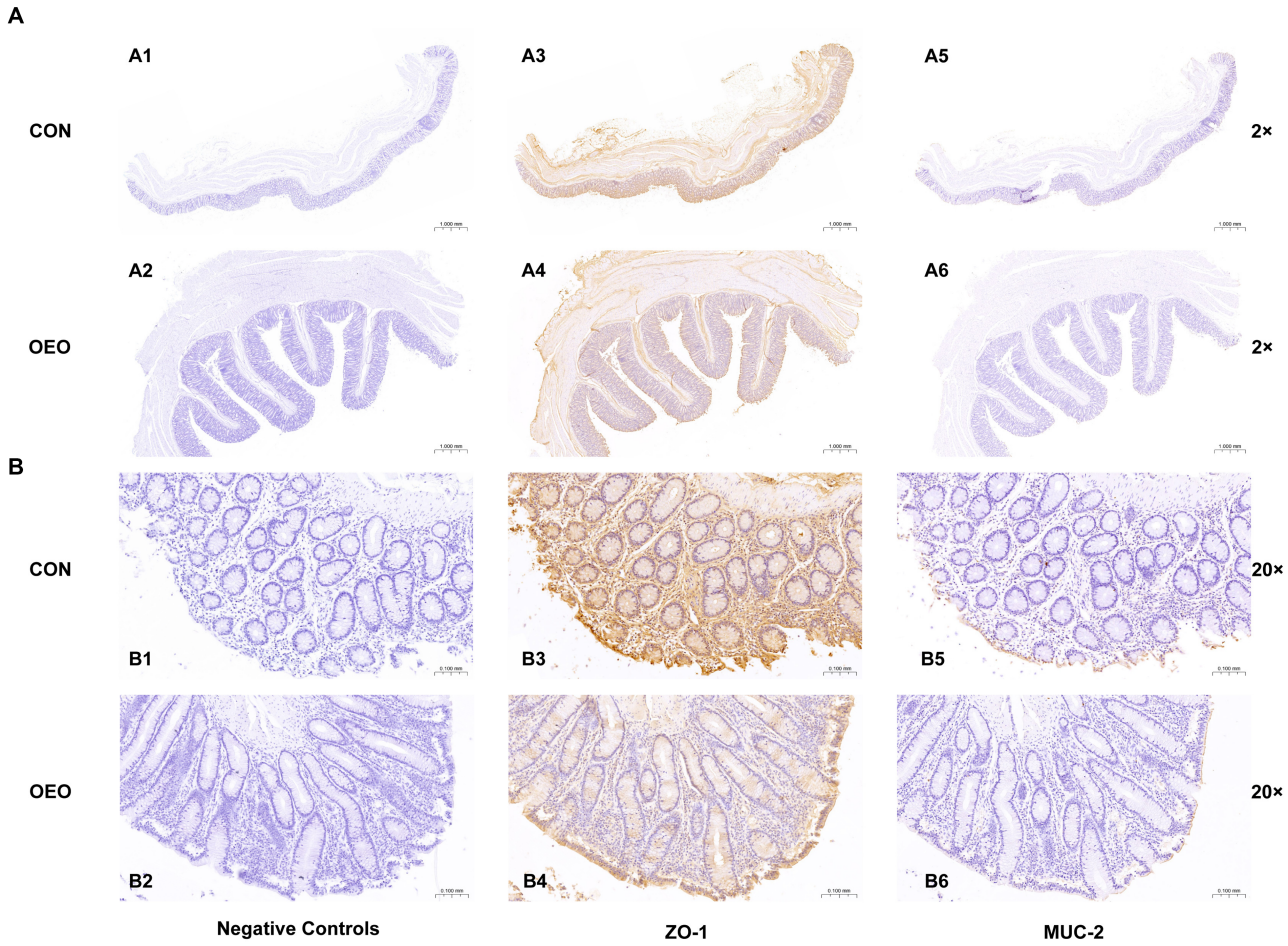
Localization of immunopositive signals for ZO-1 and MUC-2 proteins in the bull colon (2×) and (20×). **(A1–A6)**: Field of view at 2×; **(B1–B6)**: Field of view at 20×; **(A1,A2,B1,B2)**: Negative controls; **(A3,A4,B3,B4)**: Localization of ZO-l protein; **(A5,A6,B5,B6)**: Localization of MUC-2 protein; **(A1,A3,A5,B1,B3,B5)**: CON group; **(A2,A4,A6,B2,B4,B6)**: OEO group.

Positive signals for ZO-1 and MUC-2 proteins in the colonic epithelium of bulls in the CON and OEO groups were detected using immunohistochemical staining. The results revealed that the ZO-1 protein was mainly located in the tight junction intercellular ribbon region, and the MUC-2 protein was mainly located in the colonic mucous layer (Figure 3).

### 3.5 Microbial composition of colonic contents

**Figure 4:**
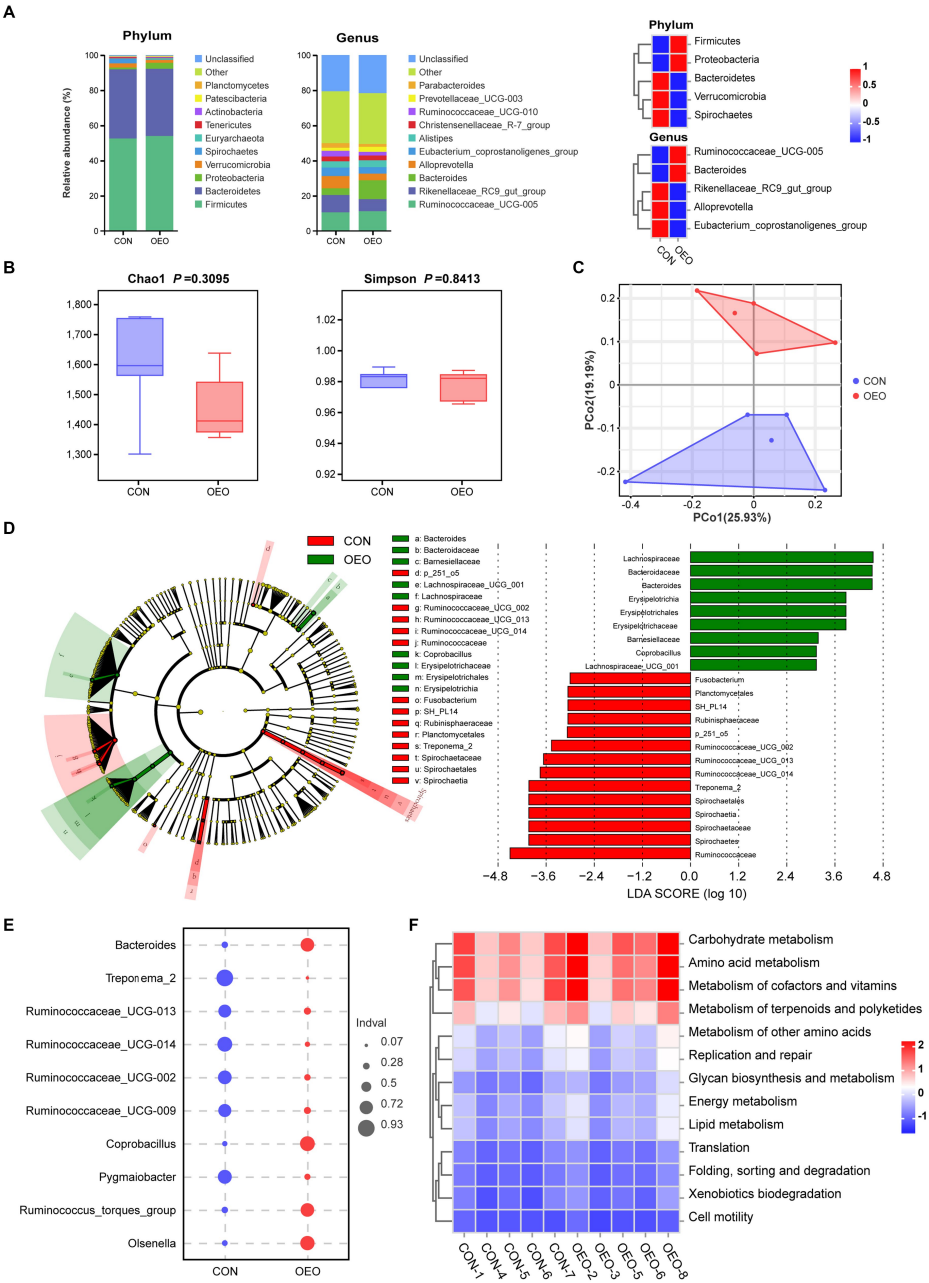
Shifts in the colonic microbiota composition of bulls after dietary supplementation with OEO. **(A)** Composition of colon microbiota of bulls at phylum and genus levels. **(B)** Alpha diversity as presented by Chao1 and Simpson indices in the colon of bulls among groups. **(C)** Principal coordinate analysis of microbial compositional profiles between the CON and OEO groups in the colonic contents of bulls. **(D)** Differences in microbial abundance between the CON and OEO groups, depicted by cladogram and LDA distribution. Total bacteria in the colon of the bulls that contribute to differences at the phylum, class, order, family, and genus levels, as analyzed using the LEfSe method (LDA score > 3). **(E)** Indicator analysis based on genus level. **(F)** Comparison of the abundances of KEGG pathways (level 2) in different groups. Red indicates a high level, and blue indicates a low level. *n* = 5 samples/group.

To further study whether OEO affects colon microbes, we performed a genetic diversity analysis of the microbial composition inside the colon using 16S rRNA amplicon sequencing. The predominant phyla were Firmicutes, Bacteroidetes, Proteobacteria, Verrucomicrobia, and Spirochaetes. The OEO group displayed an increase in Firmicutes and Proteobacteria and a decrease in Bacteroidetes, Verrucomicrobia, and Spirochaetes compared with the CON group. The predominant genera were *Ruminococcaceae_UCG-005*, *Rikenellaceae_RC9_gut_group*, *Bacteroides*, and *Alloprevotella*. The OEO group displayed increases in *Ruminococcaceae_UCG-005* and *Bacteroides* and a reduction in *Rikenellaceae_RC9_gut_group*, *Eubacterium_coprostanoligenes_group*, and *Alloprevotella* compared with the CON group (Figure 4A). We used Chao1 indices to describe richness and Simpson indices to measure species diversity across the samples, combining richness and species diversity to calculate α-diversity. The α-diversity did not differ between the CON and OEO groups (*p* > 0.05) (Figure 4B).

Principal coordinate analysis plots revealed differences in microbiota between the groups (permutational MANOVA, *R*^2^ = 0.1707, *p* = 0.027) (Figure 4C and Supplementary Table S2). Different bacteria from the domain to genus level that were specific to the OEO and CON groups were identified using the LEfSe method (Figure 4D). Among the two groups of bull colonic content samples, there were 14 dominant taxa in the CON group and 9 dominant taxa in the OEO group. Erysipelotrichia, Erysipelotrichales, Erysipelotrichaceae, *Coprobacillus*, Lachnospiraceae*Lachnospiraceae_UCG_001,* Barnesiellaceae, Bacteroidaceae, and *Bacteroides* were enriched in the OEO group. Spirochaetes, Spirochaetia, Spirochaetales, Spirochaetaceae, *Treponema_2*, Planctomycetales, Rubinisphaeraceae, *SH_PL14*, Ruminococcaceae, *Ruminococcaceae_UCG_002*, *UCG_013*, *UCG_014*, *p_251_o5*, and *Fusobacterium* were enriched in the CON group (Figure 4D and Supplementary Table S3). The results of the indicator analysis at the genus level showed that *Bacteroides*, *Coprobacillus*, *Olsenella*, and *Ruminococcus_torques_group* were indicator bacteria at the genus level in the OEO group (Figure 4E).

The potential metabolic functions of the microorganisms in different groups were predicted using the PICRUSt2 method. The OEO group promoted most of the metabolic functions of the colonic microbiota in fattening bulls, especially carbohydrate metabolism, amino acid metabolism, cofactor and vitamin metabolism, terpenoid and polyketide metabolism, and other amino acid metabolisms (Figure 4F and Supplementary Table S4).

### 3.6 Metabolome of colonic contents

**Figure 5:**
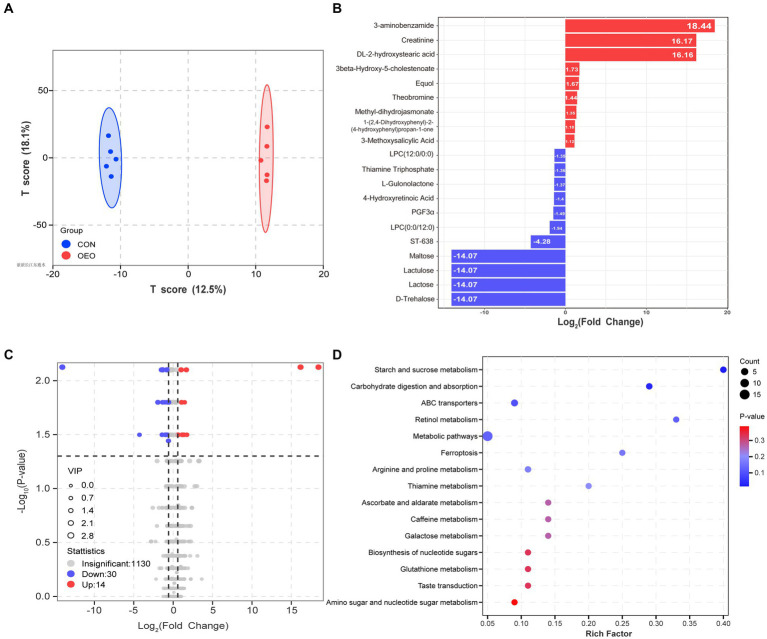
Colon metabolome changes. **(A)** OPLS-DA analysis. **(B)** Bar chart of the top 20 differential metabolites. The red color depicts significant upregulation, and the blue color depicts significant downregulation in the OEO group. **(C)** Volcano plots for the differential metabolites. **(D)** KEGG pathways (level 2) of differential metabolite enrichment.

A total of 1,174 metabolites were identified in the colon. The OPLS-DA score map showed that both groups could separate colon metabolites (*R*^2^X = 0.416, *R*^2^Y = 0.999, *Q*^2^ = 0.594; Supplementary Figure S1 and Figure 5A). After screening the relative concentrations of colon metabolites by FC (FC ≥ 1.5 and FC ≤ 0.67) and VIP (VIP ≥ 1), levels of 3-aminobenzamide, creatinine, and DL-2-hydroxystearic acid were significantly increased in the OEO group, while those of maltose, lactose, lactulose, and D-trehalose were significantly decreased (Figure 5B). The expression of 44 metabolites differed significantly between the CON and OEO groups (Supplementary Figure S2). These included 14 upregulated and 30 downregulated metabolites (Figure 5C). Analysis of the 44 differential metabolites showed that 6 were derived from carbohydrates and their metabolites, 8 were derived from amino acids and their metabolites, 5 were derived from organic acids and their derivatives, and 25 belonged to others (Supplementary Table S5).

The KEGG pathway analysis revealed that the differences mainly involved alterations in metabolic pathways (starch and sucrose metabolism, arginine and proline metabolism, and metabolic pathways), organismal systems (carbohydrate digestion and absorption), and environmental information processing (ABC transporters) (Figure 5D). Thus, dietary supplementation with OEO may have important modulatory effects on colonic carbohydrate and amino acid metabolism in fattening bulls.

### 3.7 Combined microbiome and metabolome analysis

**Figure 6:**
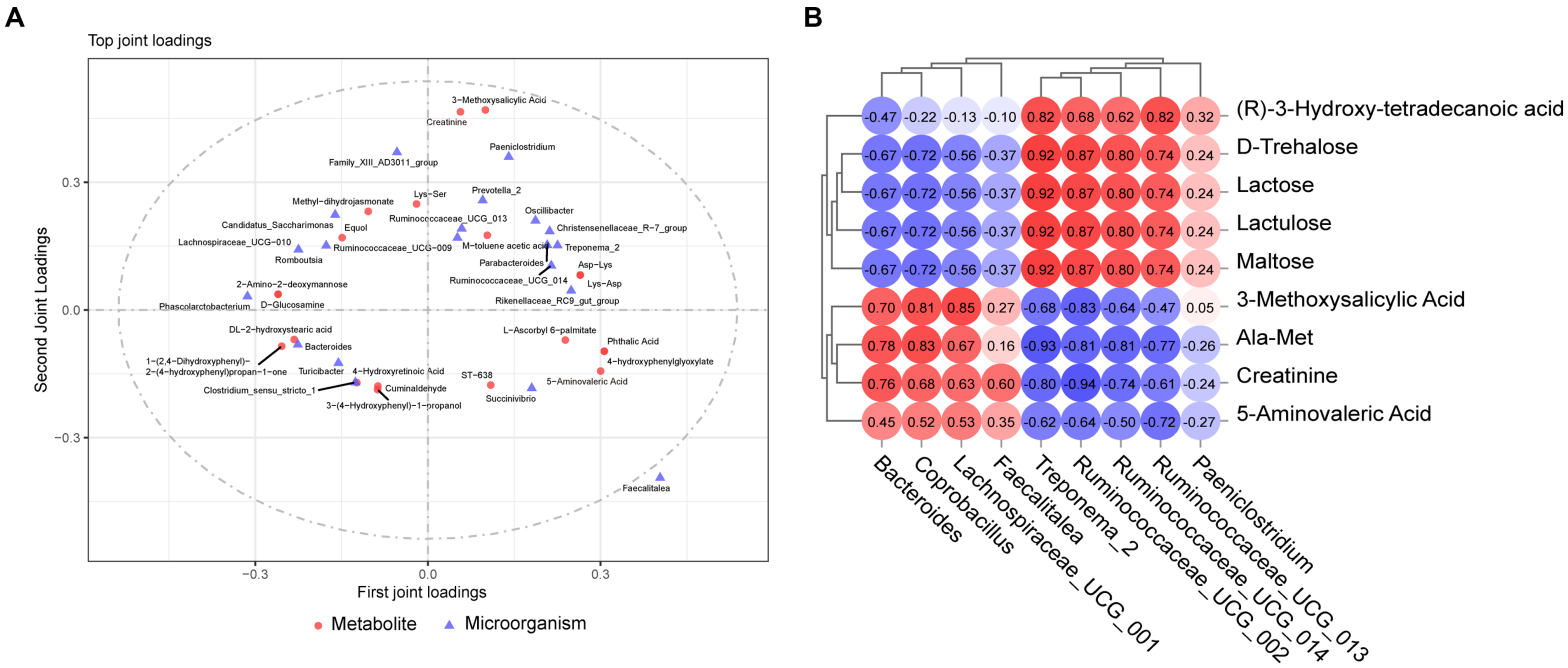
Combined microbiome and metabolome analysis. **(A)** Microbiome and metabolome O2PLS analysis. **(B)** Correlation analysis between target metabolites and microorganisms.

Microbiome and metabolome data were analyzed to examine whether there was a link between the two omics. O2PLS analysis revealed the top 20 microorganisms and metabolites with the largest linkage effects (Figure 6A and Supplementary Figure S3). Combining the results of the LEfSe analysis of the microbiome and analysis of differential metabolites in the metabolome, we screened nine different microorganisms and metabolites. To further explore the relationship between these nine metabolites and the nine microorganisms, Spearman correlation analysis was performed, and the results showed potential associations among them (Figure 6B). The relative abundances of *Bacteroides*, *Coprobacillus*, *Lachnospiraceae_UCG_001*, and *Faecalitalea* were positively correlated with maltose, lactulose, lactose, D-trehalose, and (R)-3-hydroxy-tetradecanoic acid. Furthermore, the relative abundances of *Treponema_2*, *Ruminococcaceae_UCG_002*, *UCG_013*, *UCG_014*, and *Paeniclostridium* were negatively correlated with 3-methoxysalicylic acid, Ala-Met, creatinine, and 5-aminovaleric acid, whereas *Paeniclostridium* was positively correlated with 3-methoxysalicylic acid.

### 3.8 Correlation analysis of microorganisms, metabolites, and phenotypes

**Figure 7:**
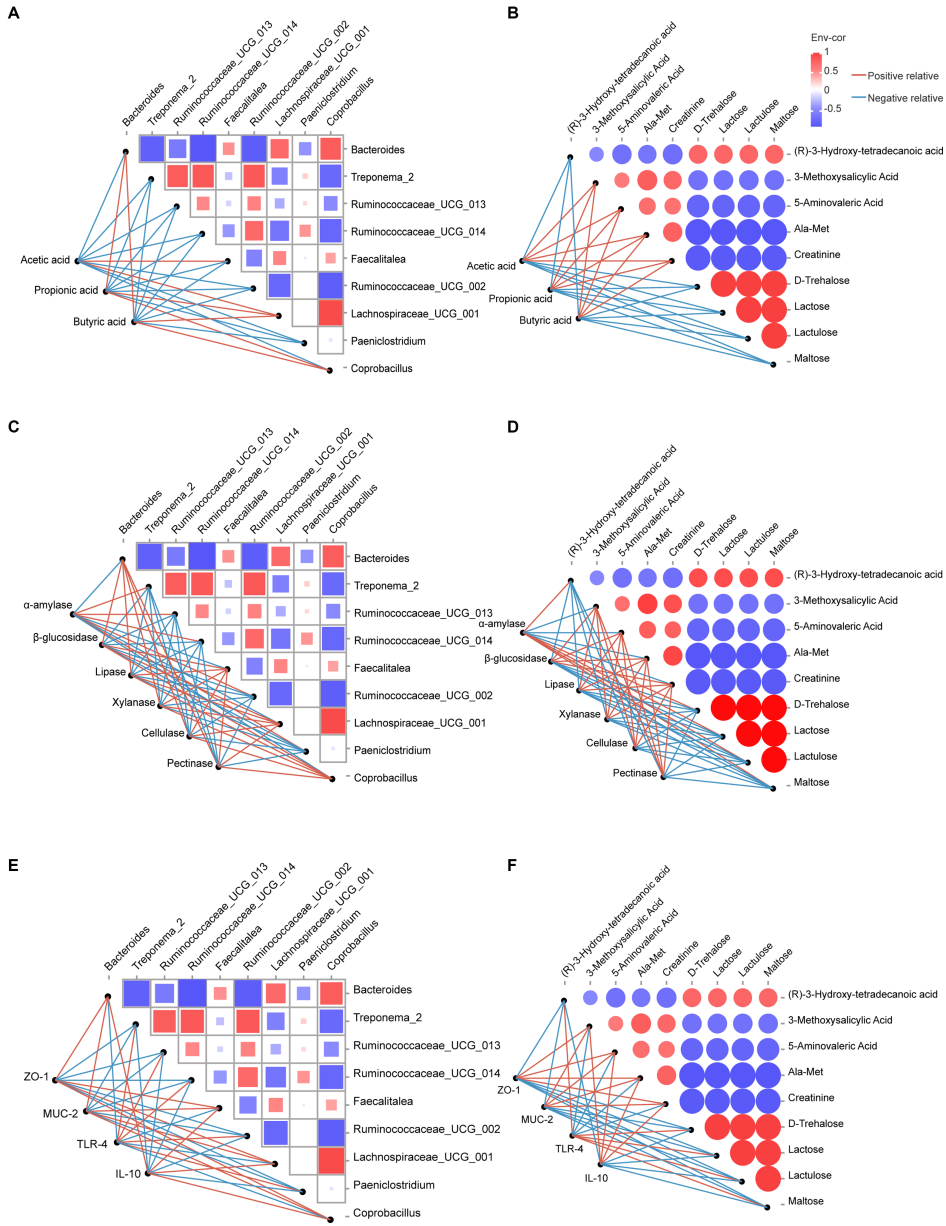
Correlation analysis between microorganisms, metabolites, and phenotypes. **(A)** Correlation analysis between microorganisms and SCFAs. **(B)** Correlation analysis between metabolites and SCFAs. **(C)** Correlation analysis between microorganisms and digestive enzyme activities. **(D)** Correlation analysis between metabolites and digestive enzyme activities. **(E)** Correlation analysis between microorganisms and intestinal barrier-related genes. **(F)** Correlation analysis between metabolites and intestinal barrier-related genes. Red lines indicate positive relative correlations, and blue lines indicate negative relative correlations.

To further explore the mechanism underlying the effect of dietary OEO on the colonic barrier function of fattening bulls, we performed Spearman’s correlation analyses of the relationships among microorganisms, metabolites, and phenotypes. The results showed that acetic acid levels were positively correlated with both *Faecalitalea* and *Lachnospiraceae_UCG_001*, and propionic and butyric acid levels were positively correlated with *Bacteroides*, *Lachnospiraceae_UCG_001*, and *Coprobacillus* (Figure 7A). All four metabolites (3-methoxysalicylic acid, 5-aminovaleric acid, Ala-Met, and creatinine) were positively correlated with acetic acid, propionic acid, and butyric acid (Figure 7B).

Lipase, β-glucosidase, xylanase, cellulase, pectinase activity, and the mRNA expression of *ZO-1*, *MUC-2*, and *IL-10* were positively correlated with *Lachnospiraceae_UCG_001*, *Bacteroides*, *Faecalitalea*, *Coprobacillus* (Figures 7C,E), 5-aminovaleric acid, 3-methoxysalicylic acid, Ala-Met, and creatinine (Figures 7D,F) and negatively correlated with *Treponema_2*, *Ruminococcaceae_UCG_002*, *UCG_013*, *UCG_014*, *Paeniclostridium* (Figures 7C,E), maltose, lactulose, lactose, D-trehalose, and (R)-3-hydroxy-tetradecanoic acid (Figures 7D,F). However, α-amylase activity and mRNA expression of *TLR-4* showed opposite trends.

## 4 Discussion

The growth performance, slaughter performance, and carcass characteristics were previously published (Fan et al., 2022). Compared with CON group, the average daily gain, body weight, and carcass weight in OEO group were increased by 20.5, 15.5, and 15.7%, respectively, and the fattening effect was significant. In this study, we assessed the effects of dietary supplementation with OEO on the hindgut microbiome and metabolome, both of which play a crucial role in colonic physiology and barrier function in fattening bulls. We found that OEO administration led to an increased abundance of *Bacteroides* and *Lachnospiraceae_UCG_001*. Further, it also stimulated the production of microbial metabolites through carbohydrate and amino acid fermentation, as evidenced by elevated luminal concentrations of acetate, propionate, butyrate, 5-aminovaleric acid, and creatinine. Additionally, OEO supplementation significantly impacted the maintenance of the functional integrity of the intestinal barrier. This was evident from the observed effects on colonic histomorphology, immunohistochemistry, digestive enzyme activity, and relative mRNA expression of intestinal barrier-related genes. These findings suggest that the addition of OEO positively impacts the integrity of the intestinal barrier in fattening bulls, and this effect is mediated by enhancements in the colonic microbiota and metabolites.

The symbiotic relationship between the gastrointestinal tract of the host and its resident microbiota plays a crucial role in the maintenance of physiological balance in the organism (Ma et al., 2022). Consequently, maintaining gut homeostasis is essential for protecting the intestinal health and barrier function of the host. The intestinal microflora is closely related to host metabolic conditions and dietary composition (Rastelli et al., 2019), and it directly contributes to the formation of the intestinal epithelial barrier, which protects the intestinal tissue from pathogenic bacteria and toxins (De Santis et al., 2015). Plant essential oils modulate ruminant production performance, primarily by influencing nutrient utilization. The possible mechanisms by which plant essential oils affect the digestion rate of nutrients are mainly enzyme stimulation and cellular and microbial mechanisms. Plant essential oils are a type of feed additive, and their addition to feed significantly affects the abundance of intestinal flora in cattle (Clemmons et al., 2019). OEO alters epithelial development and microbiota composition in beef cattle to improve rumen digestion (Zhang et al., 2021) and enhances the intestinal barrier integrity of the jejunum and ileum in sheep (Jia et al., 2022). Given these considerations, we chose OEO as a feed additive for this study to investigate its potential mechanisms for influencing the microflora and intestinal barrier function in the colon of fattening bulls.

Ruminants have a richer gut microbiota than monogastric animals. The composition of gastrointestinal microorganisms in ruminants varies due to genetics, environment, and diet; however, similarities exist among the dominant bacteria. Regarding the microbial composition in ruminant colons, our study found that Firmicutes, Bacteroidetes, and Proteobacteria were the predominant phyla in the colonic contents across all samples. This result is in line with earlier findings in goats (Wang et al., 2020), Hu sheep (Lin et al., 2021), and dairy cows (Yuchao et al., 2023). Further, at the genus level, the relative abundances of *Bacteroides*, *Coprobacillus*, *Lachnospiraceae_UCG_001*, and *Faecalitalea* were enhanced in the OEO group. Some of these results were surprising. Members of the genus *Bacteroides* are major players in sustaining the microbial food web of the gut and are considered potential colonizers of the colon (Zafar and Saier, 2021). Additionally, *Bacteroides* competes with pathogens for host-derived amino acids and monosaccharides and produces SCFAs, thereby countering pathogens directly (Bornet and Westermann, 2022). *Coprobacillus* is associated with butyrate production in mouse intestines (Ye et al., 2018) and is considered a potential probiotic strain (do Prado et al., 2021). Multiple strains within the Lachnospiraceae bacterial family possess the ability to metabolize carbohydrates, resulting in the production of butyrate and other SCFAs (Zhang et al., 2023). *Faecalitalea,* a member of the family Erysipelotrichaceae, has been proposed as a key butyrate producer (Zhou et al., 2021). These results suggest that OEO does indeed modulate the abundance of intestinal bacteria, positively affecting the host. The mechanisms by which these key microorganisms maintain homeostasis in the internal intestinal environment while influencing barrier function require further exploration, in conjunction with microbial metabolites.

Colonic microbiota can ferment undigested chow and host-generated and microorganism-produced endogenous compounds, yielding various metabolites (Zhao et al., 2022). The maintenance of intestinal homeostasis in the host is primarily regulated by the intestinal flora or the SCFAs produced by them (Akhtar et al., 2022). The current investigation revealed significant correlations between the intestinal microbiota and metabolites, as seen by the integration of microbiome and metabolome studies. These findings demonstrate notable changes in the concentrations of SCFAs, 5-aminovaleric acid, 3-methoxysalicylic acid, creatinine, and several other metabolites. The role of the intestinal microbiome in host health is predominantly mediated by SCFA metabolites, which are the main metabolites produced by specific intestinal microbiomes that ferment resistant starch and dietary fiber (Yao et al., 2022). The colonic microbiota of ruminants primarily degrades carbohydrates in the feed and produces SCFAs to provide energy and nutrients to the host. In bacteria, SCFAs are waste products that are essential for balancing redox equivalent formation under anaerobic conditions (van Hoek and Merks, 2012). These metabolites are represented by organic acids, such as acetate (C2), propionate (C3), and butyrate (C4), which are composed of less than six carbon atoms. Microbiota-derived SCFAs play dual roles in the host and the pathogen (Mirzaei et al., 2022). We speculated that OEO promotes SCFA production by modulating the abundance of certain beneficial bacteria, thereby positively affecting colonic physiology and barrier function in fattening bulls. The OEO group showed significantly higher concentrations of propionic and butyric acids, which may have beneficial effects, as butyric acid is preferentially used as an energy source by the intestinal mucosa (Louis and Flint, 2017). Moreover, butyric acid can act as a signaling molecule to regulate various functions in the host, such as exerting a modulatory effect on the intestinal immune system and inflammation (Yao et al., 2022). Propionic acid acts as an important gluconeogenic precursor produced in ruminants and has a hormone-like first-messenger function (Duscha et al., 2020). Acetic acid is generated as a result of the fermentation process carried out by several bacterial strains (Liu et al., 2018). A large number of complex carbohydrates are continuously broken down by bacteria within the phylum Bacteroidetes (van der Hee and Wells, 2021). The main product of *Bacteroidetes* fermentation is propionate, whereas that of Firmicutes is butyrate (Ma et al., 2022). No single bacterium hydrolyzes all nutritional substrates; hence, no unique bacterial fermentation of carbohydrates produces the three SCFAs. The type and distribution of SCFAs in the gut represent metabolic cooperation between various bacterial species. Microbes that efficiently produce SCFAs are generally considered beneficial (Kim, 2023). *Bacteroides*, *Coprobacillus*, *Faecalitalea*, and *Lachnospiraceae_UCG_001*, which increased in abundance in the colonic flora after OEO supplementation, were considered beneficial bacteria. Overall, SCFAs are both metabolites of intestinal flora and regulators of intestinal flora homeostasis. SCFAs play essential roles in regulating the intestinal tract’s physical, chemical, microbial, and immune barriers by influencing pH and mucus production and providing energy to intestinal epithelial cells. They also contribute to the construction of nonspecific defense barriers, thereby maintaining intestinal barrier function. In our study, microbial metabolites (represented by SCFAs) bridged the connection between microbes and intestinal barrier function.

In addition to SCFAs, other microbial metabolites play important roles in intestinal physiology and barrier function. For example, the GABA analog 5-aminovaleric acid can increase brain glutamine concentrations (Dhaher et al., 2014). Additionally, 3-methoxysalicylic acid is a derivative of salicylic acid with anti-inflammatory and antioxidant properties (Reszka et al., 2005). We also observed decreased maltose, lactulose, lactose, and D-trehalose levels in the OEO group. This indicated that the microbes in this group utilized carbohydrates to a greater extent and fermented them more fully. The main substrates for bacterial fermentation and SCFAs production are inulin, cellulose, guar gum, pectin, and resistant starch (Venegas et al., 2019). Monogastric animals digestive system lacks enzymes to digest complex polysaccharides, such as pectins, xylan or celluloses, consequently reaching the colon with their intact structure, and subsequently fermented by colonic bacteria (Carlson et al., 2017). However, in general, ruminants’ digestive enzymes are more likely to break down plant fiber, which is vital to animal health (Zhang et al., 2021). After intestinal digesta is digested and absorbed in the small intestine, the residual portion enters the hindgut (Zhao et al., 2022). Where the small intestinal digestive enzymes that come with the chow are fully mixed with the large intestinal fluid. Digestive enzymes in the colon play a limited role in chemical digestion, and mainly rely on microorganisms for biotic digestion. In addition, digestive enzyme activity is part of the study of the intestinal chemical barrier. Even though digestive enzymes have a limited function in colonic digestion, our study cannot selectively ignore their other function. Phenolics (especially thymol and carvacrol as active components of OEO) improve the activity of intestinal digestive enzymes in chickens (Hashemipour et al., 2013). Research on adding OEO to sheep feeds revealed similar outcomes (Jia et al., 2022). Moreover, the pH of the colonic digesta exhibited a reduction as a result of elevated quantities of acetate, propionate, and butyrate in the colonic digesta, therefore generating a mildly acidic milieu. Colonic fermentation of fiber to SCFAs decreases pH levels, increases fecal acidification, and increases the growth and diversity of the gut microbiota taxa (Portincasa et al., 2022). Fouhse et al. (2016) demonstrated that a decrease in intestinal pH can, to a certain extent, increase the activity of digestive and microbial enzymes in the intestine, inhibit the proliferation of pathogenic bacteria, and reduce susceptibility to disease. Therefore, we believe that OEO supplementation enhances microbial carbohydrate fermentation and reduces colonic pH, which is beneficial for strengthening the intestinal barrier function of fattening bulls.

The intestinal barrier, which includes mechanical, chemical, immune, and microbial barriers, is crucial for the host to resist invasion by foreign pathogens. Any factor that disrupts the integrity of the intestinal barrier can lead to host metabolic dysfunction and affect intestinal health, thereby adversely affecting livestock health and production performance (Ghosh et al., 2021). The intestinal morphology and tight junctions are important parameters that reflect the intestinal physical barrier. As the mucosal lining of the gastrointestinal tract directly interfaces with the external gut lumen, which is heavily populated with bacteria, viruses, fungi, archaea and protists, it is also an important physical barrier to invading pathogens (Rogers et al., 2023). Compared to the complex squamous epithelial structure of the rumen, the hindgut consists of only a single layer of epithelial cells, making the hindgut barrier of ruminants more susceptible to damage during feeding of high grain diets (Petri et al., 2021). Furthermore, the surface of the hindgut mucosa was smooth, without folds and intestinal villus, which also emphasizes its limited absorptive capacity. The findings of our study indicate that the administration of OEO resulted in enhanced intestinal morphology and elevated expression of tight junction proteins in the intestines of fattening bulls. These observations imply that OEO may contribute to the improvement of the intestinal physical barrier. Moderate concentrations of butyrate promoted the relative expression of *Occludin* and *ZO-1* mRNA in rat IPEC-J2 cells (Ma et al., 2012). Moreover, propionic acid increased the expression of the intestinal tight junction proteins ZO-1 and Occludin (Tong et al., 2016). The intestinal chemical barrier primarily consists of a mucus layer that covers the intestinal epithelial cells. MUC is the dominant molecule in the mucosal layer and is mainly secreted by goblet cells. Intestinal bacteria may affect mucus production and quality (Jakobsson et al., 2015). Disturbances in microbiota affect the development of colon goblet cells (Grey et al. 2022). SCFAs upregulate the expression of *MUC-1* and *MUC-2* in the intestine, thereby strengthening the intestinal chemical barrier function (Sun et al., 2017). Appropriate concentrations of butyrate were found to significantly improve the barrier function of human colonic epithelial cells by increasing the level of MUC-2, whereas excessive concentrations of butyrate decreased the barrier function (Nielsen et al., 2018). These findings are consistent with our findings. We found that OEO upregulated the expression of genes associated with the intestinal chemical barrier by mediating the production of additional SCFAs by the colonic microbiota.

The study shows that feeding cattle more than 44.1% concentrate in the diet was associated with gastrointestinal dysbiosis and an increase in the risk of systemic inflammation (Zebeli et al., 2012). To reduce the incidence of intestinal inflammation and minimize its damage, a measure used in production is to gradually increase the proportion of grains in the diet over a period of time in order to achieve a transition from a roughage-based diet to a high grains diet for bulls. At the same time, harmful metabolites produced by dysbiosis of the intestinal flora can endanger the intestinal barrier and immune function. Enteric infections and inflammations are often typically manifest within the intestines, causing damage to the intestinal lining, including the collapse or displacement of structural integrity mechanisms, and microbial dysbiosis (Rogers et al., 2023). Endotoxins, such as lipopolysaccharide, are released into the intestinal digesta (Khafipour et al., 2016). These releases increase the concentration of luminal endotoxins and contribute to gut epithelial damage (Zhao et al., 2023). Existing studies have shown that gastrointestinal tract-derived lipopolysaccharide increases the expression of inflammatory cytokines, such as TLR-4, IL-1β and TNF-α, in the intestines and blood by regulating a large number of immune genes, eventually leading to a state of systemic inflammation (Monteiro and Faciola, 2020). In the fattening of bulls, the attempt to minimize the inflammatory effects on the host from the production of endotoxins of intestinal origin must rely on the relative stability of the microbial flora in the intestine and the barrier function of the intestinal immune cells. The intestinal immune barrier plays a crucial role in the ability of the host to resist invasion by pathogenic bacteria. The crosstalk between gut microbiota and the immune system is intricate and is partially dependent on gut microbial metabolites (Yang and Cong, 2021). Additionally, due to differences in epithelial structure, the gut is more susceptible to the effects of contents than the rumen. The interactions between intestinal epithelial cells, intestinal immune tissues and commensal microorganisms form a complex ecosystem. At homeostasis, this ecosystem suppresses and balances the mucosal immune response to highly immunogenic intestinal contents, thereby avoiding uncontrolled inflammatory responses (Jamwal et al., 2020). Intestinal epithelial cells are able to produce a variety of inflammatory cytokines, including IL-6, TNF-α, and IL-1β (Cosin-Roger et al., 2017). In their study of inflammatory bowel diseases in humans, the researchers found that the expression of the inflammatory cytokines IL-6, TNF-α, and IL-1β were, respectively, upregulated 2.266, 0.962, and 3.468 fold, in both Crohn’s disease and ulcerative colitis compared to healthy individuals (Leppkes and Neurath, 2020). Toll-like receptors (TLRs), which are representative pattern recognition receptors, can facilitate the recognition of microbial molecules to stimulate immune responses (Round et al., 2011). TLR-4 can detect lipopolysaccharide, a prominent constituent of the outer membrane of gram-negative bacteria. The efficacy of OEO in inhibiting the expression of *TLR-4*, *IL-1b*, *TNF-a*, and *IFN-γ* has been demonstrated through the TLR4-mediated signaling pathway (Feng et al., 2021). Furthermore, OEO exhibited a downregulatory effect on the mRNA expression of *TNF-a* and *IL-6* in rats (Wei et al., 2015). Metabolites from microorganisms represented by SCFAs can not only promote the differentiation and function of immunosuppressive cells but also inhibit the inflammatory cells, together maintaining the gut and systemic immune homeostasis of the individuals (Wang et al., 2023). The immunoregulatory capacity of SCFAs, which refers to their ability to maintain a balance between anti-inflammatory and proinflammatory responses, is influenced by the composition of the intestinal flora (Kim, 2021). In the current investigation, it was observed that the proinflammatory cytokines TLR-4, TNF-α, and IL-1β significantly decreased, while the anti-inflammatory cytokine IL-10 significantly increased in the colon of the OEO group. These findings suggest enhanced functionality of the intestinal immune barrier. This phenomenon may be explained by the microbial synthesis of SCFAs, which can induce cell proliferation or differentiation by influencing the activation of cellular receptors. Furthermore, SCFAs may function as histone deacetylase inhibitors, thereby affecting intestinal immunity.

The intestinal microbial barrier is widely recognized as a stable microecosystem consisting of normal microbial flora residing in the intestinal tract. This microecosystem is of great importance as it significantly contributes to the development and regulation of immune functions (Lee et al., 2022). Our results showed that OEO did not disrupt the homeostasis of the internal intestinal environment. Moreover, it enriched the number of beneficial bacteria and inhibited the growth of harmful bacteria to some extent. One potential explanation for this phenomenon is that microbial metabolites primarily exert their inhibitory effects on pathogenic bacteria through various mechanisms, such as the release of H^+^ ions, which leads to a reduction in intestinal pH. Additionally, microbial metabolites may compete for energy resources, generate antimicrobial peptides, and impede the biosynthesis of harmful bacteria. Collectively, these actions contribute to the establishment of a balanced intestinal microecology. However, some mechanisms require further investigation.

## Data availability statement

The datasets presented in this study can be found in online repositories. The names of the repository/repositories and accession number(s) can be found at: NCBI - PRJNA995187.

## Ethics statement

The animal studies were approved by Animal Care Committee of Gansu Agricultural University (permit no. GSAU-Eth-AST-2022-035). The studies were conducted in accordance with the local legislation and institutional requirements. Written informed consent was obtained from the owners for the participation of their animals in this study. Written informed consent was obtained from the individual(s) for the publication of any potentially identifiable images or data included in this article.

## Author contributions

YM: Conceptualization, Writing – original draft, Writing – review & editing. JS: Data curation, Investigation, Writing – review & editing. LJ: Methodology, Supervision, Writing – review & editing. PH: Methodology, Writing – review & editing. YW: Investigation, Writing – review & editing. XZ: Methodology, Writing – review & editing. YH: Formal analysis, Writing – review & editing. QC: Supervision, Writing – review & editing. ZZ: Supervision, Writing – review & editing. YD: Methodology, Writing – review & editing. MX: Validation, Writing – review & editing. ZL: Conceptualization, Data curation, Funding acquisition, Project administration, Resources, Writing – review & editing.

## Footnotes

^1^ http://www.metware.cn/

^2^ https://sciex.com.cn/
