# Supplementary figures and images for: Oregano essential oil modulates colonic homeostasis and intestinal barrier function in fattening bulls
Source: Front Microbiol. 2023 Dec 5;14:1293160. doi: 10.3389/fmicb.2023.1293160 (PMC10728825; doi:10.3389/fmicb.2023.1293160)

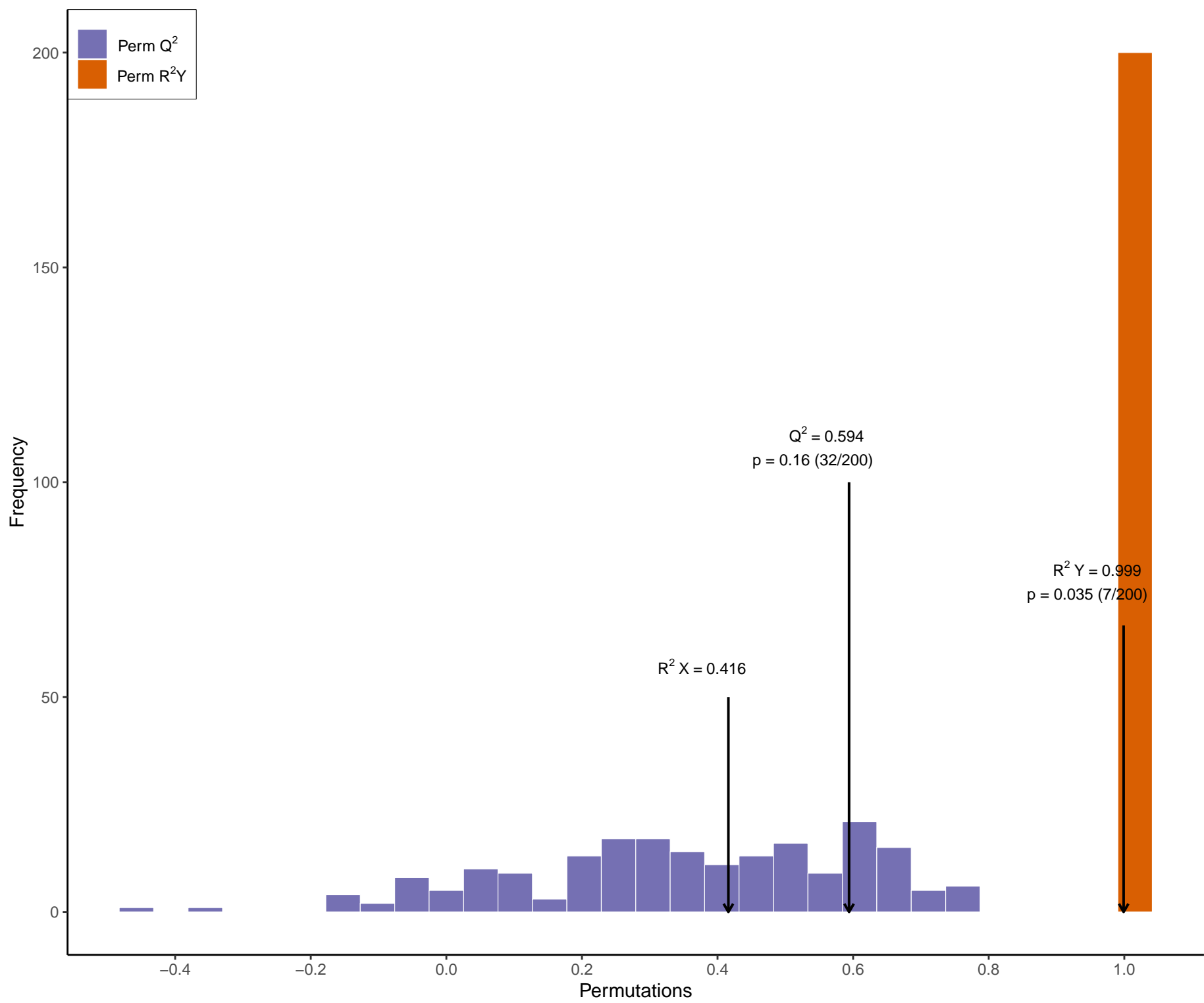

Supplement: Supplementary file 1 [file Data_Sheet_1.ZIP › Supplementary Material/Supplemental Fig. S1_Reliability of the OPLS-DA model.pdf]

Violin Plot of Raw Values

Raw Intensity

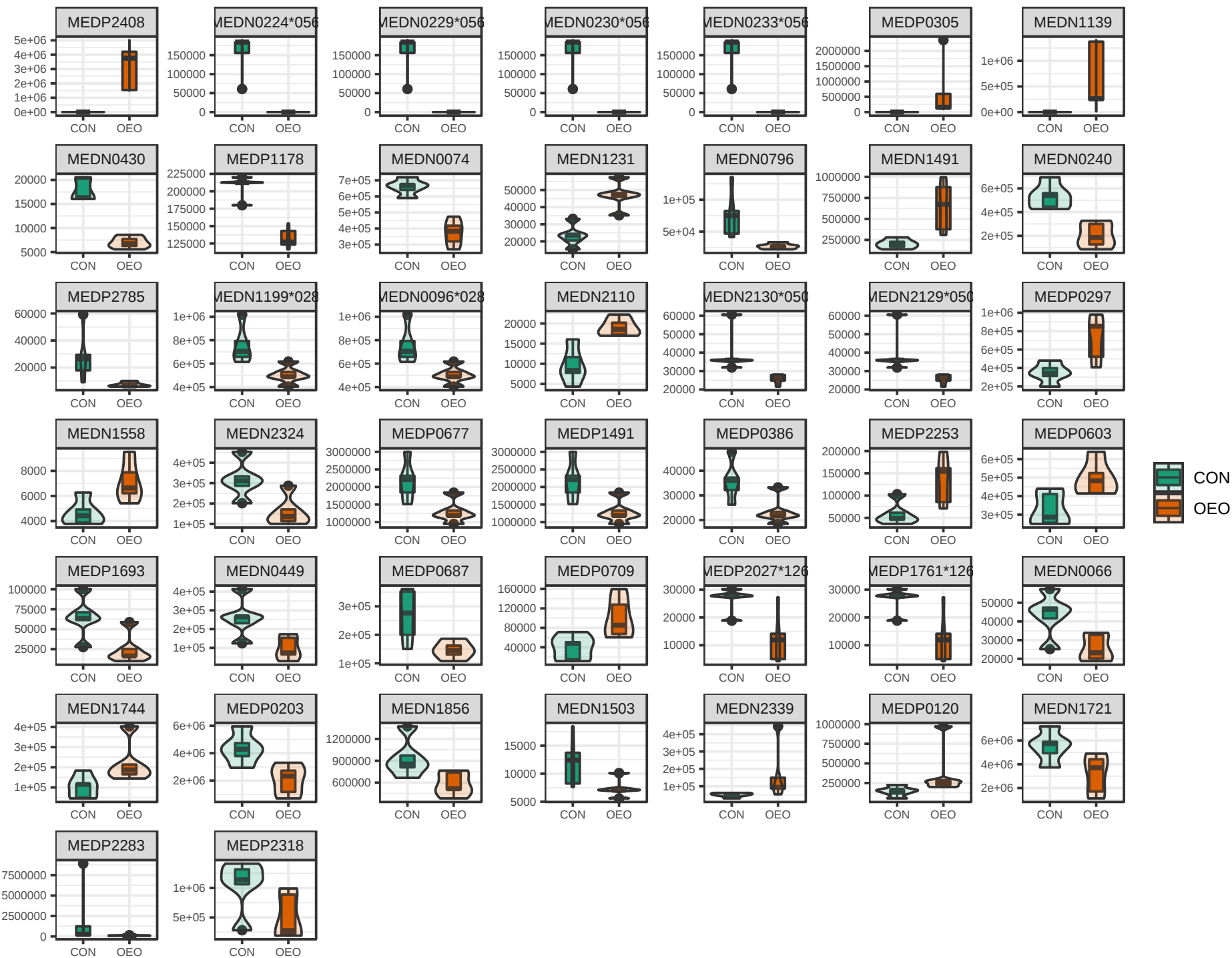

Supplement: Supplementary file 1 [file Data_Sheet_1.ZIP › Supplementary Material/Supplemental Fig. S2_Significantly different metabolites.pdf]

# X joint loadings

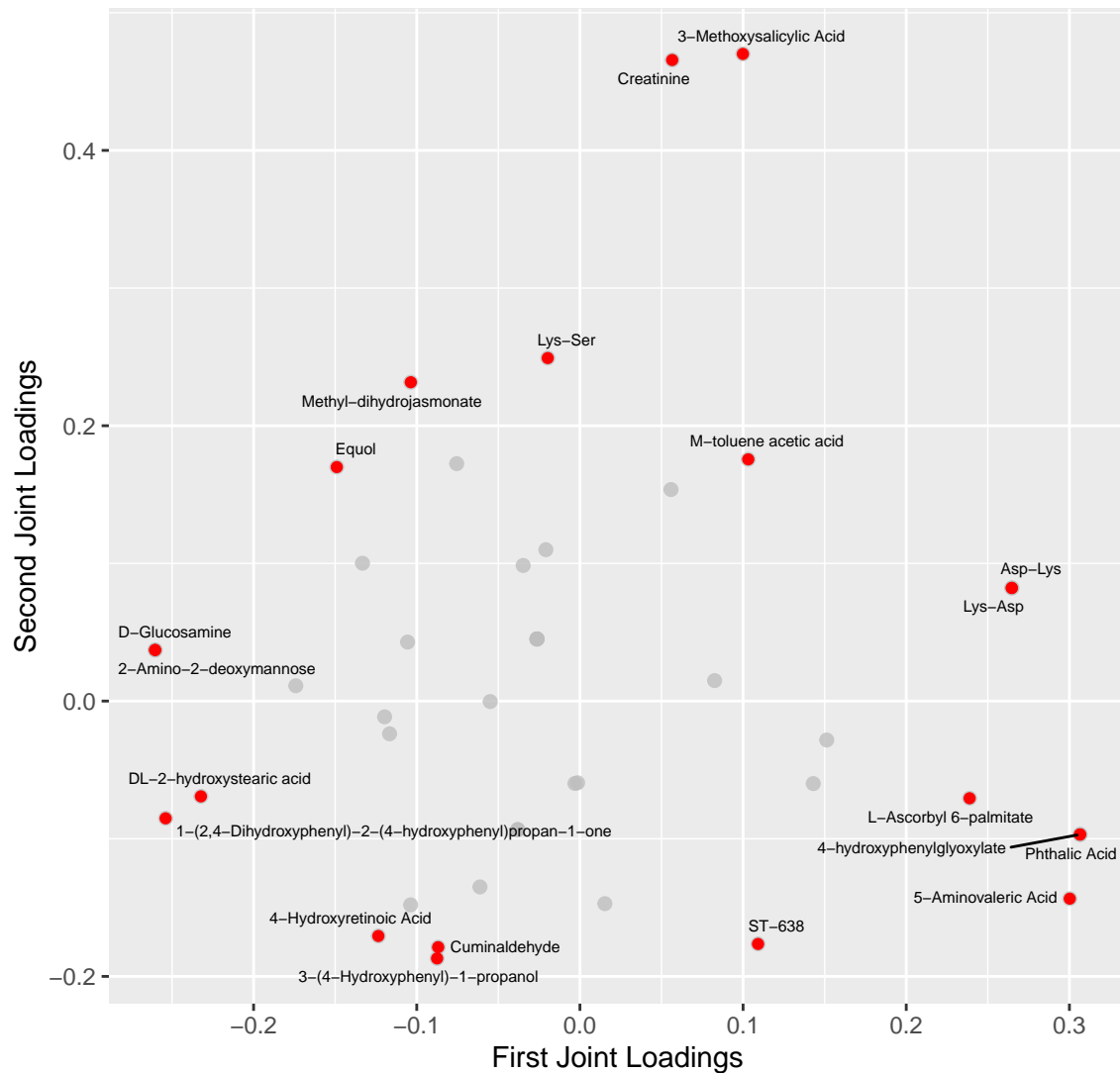

# Y joint loadings

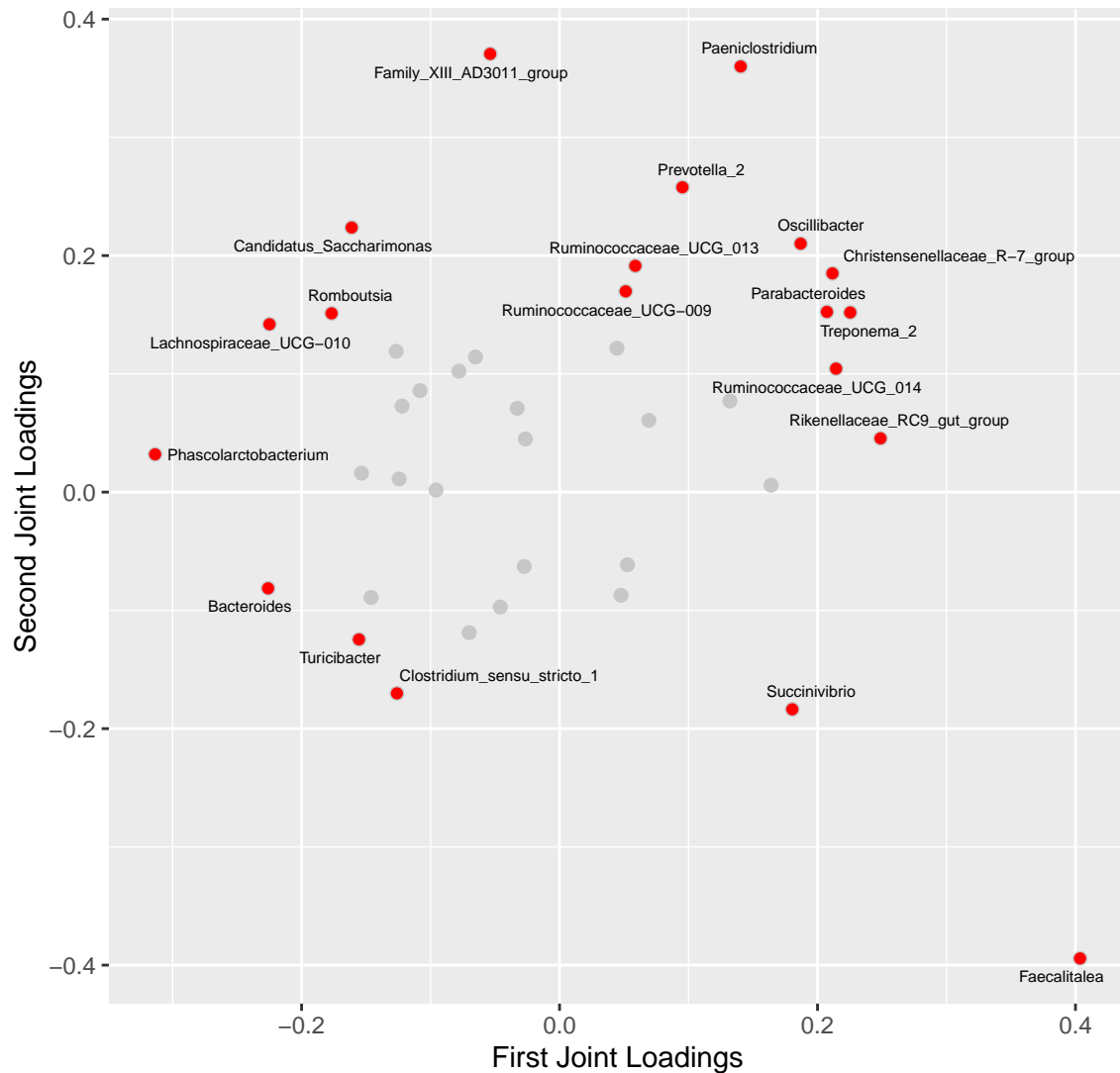

Supplement: Supplementary file 1 [file Data_Sheet_1.ZIP › Supplementary Material/Supplemental Fig. S3_O2PLS loadings plot.pdf]
